# Disentangling the ecosystem service ‘flood regulation’: Mechanisms and relevant ecosystem condition characteristics

**DOI:** 10.1007/s13280-022-01708-0

**Published:** 2022-02-25

**Authors:** Ágnes Vári, Zsolt Kozma, Beáta Pataki, Zsolt Jolánkai, Máté Kardos, Bence Decsi, Zsolt Pinke, Géza Jolánkai, László Pásztor, Sophie Condé, Gabriele Sonderegger, Bálint Czúcz

**Affiliations:** 1 Centre for Ecological Research, Lendület Ecosystem Services Research Group, Alkomány út 2-4, Vácrátót, 2163 Hungary; 2 Department of Sanitary and Environmental Engineering, Budapest University of Technology and Economics, Műegyetem rkp. 3, Budapest, 1111 Hungary; 3 Department of Civil Engineering, University of Debrecen, Ótemető u. 2-4, Debrecen, 4028 Hungary; 4 Department of Physical Geography, Eötvös Loránd University, Pázmány Péter sétány 1/C, Budapest, 1117 Hungary; 5 Institute for Soil Sciences, Centre for Agricultural Research, Budapest, 1022 Hungary; 6 European Topic Centre on Biological Diversity, Muséum National d’Histoire Naturelle, 57 rue Cuvier, 75231 Paris Cedex 05, France; 7 Umweltbundesamt, Spittelauer Lände 5, 1090 Wien, Austria

**Keywords:** Ecosystem characteristics, EU Flood Directive, Flood mitigation, Flood prevention

## Abstract

Riverine floods cause increasingly severe damages to human settlements and infrastructure. Ecosystems have a natural capacity to decrease both severity and frequency of floods. Natural flood regulation processes along freshwaters can be attributed to two different mechanisms: flood prevention that takes place in the whole catchment and flood mitigation once the water has accumulated in the stream. These flood regulating mechanisms are not consistently recognized in major ecosystem service (ES) classifications. For a balanced landscape management, it is important to assess the ES flood regulation so that it can account for the different processes at the relevant sites. We reviewed literature, classified them according to these mechanisms, and analysed the influencing ecosystem characteristics. For prevention, vegetation biomass and forest extent were predominant, while for mitigation, the available space for water was decisive. We add some aspects on assessing flood regulation as ES, and suggest also to include flood hazard into calculations.

## Introduction

The frequency of severe floods as well as the damages they cause have been increasing in the last decades globally (Jongman et al. 2012; European Environment Agency (EEA) 2019). According to projections, they are going to further rise with our climate changing and precipitation events getting more extreme both in terms of intensity and frequency in many parts of the world (Frei et al. 2006; IPCC 2014; EEA 2019). The increase in flood frequency and severity is generally associated with a deteriorating ecosystem quality resulting from more intense anthropogenic influences: intense land management, consumption and conversion of land, land take in flood-prone areas, increasing exposure to flood hazard, river straightening, as well as (hydromorphological) processes such as imbalances in sediment transport in the main channel and along the floodplains (Józsa et al. 2014; Sofia and Nikolopoulos 2020). River straightening and flood control infrastructure has been extended since the mid-nineteenth century, and had to be upgraded with rising frequency of floods in the past decades (Kiss et al. 2019). The efficiency of artificial flood control has to be questioned in the light of increasingly extreme weather issues and rising defence costs (Koncsos 2011). It is more and more the natural flood regulation capacity of the landscapes that needs to be revitalized and that we need to focus on.

Generally, a precipitation induced flood can be loosely defined as the overflow of water—resulting from both surface and sub-surface runoff as well as from water spilling from the river—on land which is usually dry (Kron 2005). Such rise of the water level is considered a temporary event and can occur in creeks, rivers, canals, lakes or within wetlands (but we deliberately exclude here coastal floods). The term ‘flood hazard’ itself can be seen as an anthropocentric term (see Brauman et al. 2007; Koncsos 2011) as it generally describes the conflict between human made infrastructure, including land use, and the extent of inundation. The consequences of floods can be both negative but also positive (Brauman et al. 2007; Wantzen et al. 2016; Tomscha et al. 2017) and can vary greatly depending on the location, duration, depth and speed of the flooding events, as well as the vulnerability and the economic value of the locations they affect (Kron 2005).

The concept of ecosystem services (ES) (Daily 1997; Schröter et al. 2017) was developed to reflect on environmental issues arising from deteriorating environmental quality, unsustainable use of resources and decline of natural habitats. This intuitive concept inspired the evolution of increasingly formalized and standardized ‘ES assessments’ (Steger et al. 2018) offering a structured quantification of the dependencies of human societies on natural ecosystems for smarter land use and policy decisions. Multiple aspects can be taken into account—weighted by stakeholders—as multifunctionality indices, during decision making or in decision support systems. In order to be able to assess the ecosystem service flood regulation correctly within an ES framework, it is important to identify the key elements determining the occurrence and severity of flood events, and link them to the ES framework in a consistent way (Finisdore et al. 2020). It is also important to formulate plain, easy-to-communicate messages regarding land use and water management. The messages should be based on the performed assessments, and in line with sustainability criteria, i.e. incorporate nature-based solutions.

**Fig. 1:**
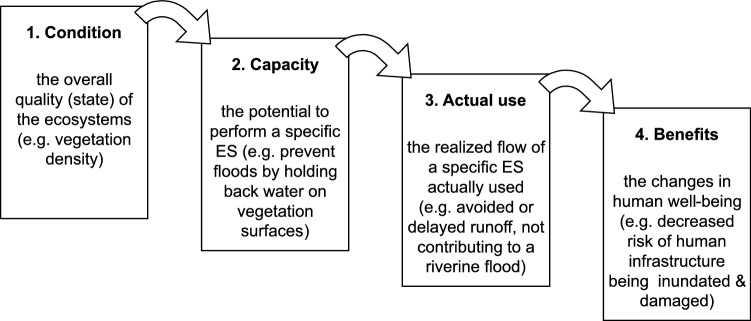
Flow of ecosystem services from ecosystems to humans in the cascade framework adapted from Czúcz et al. (2020) (originally based on Haines-Young et al. (2010))

Most of the recent ES assessments rely on some form of the ES cascade framework (Haines-Young and Potschin 2010; Potschin-Young et al. 2018; Heink and Jax 2019). This framework provides a general model of the flow of ES from the ecosystems towards the society in several key steps (‘cascade levels’, see Fig. 1). These steps identify the critical points in the ES flow, where the relevant actions of enablement and appropriation take place (Spangenberg et al. 2014; Heink a Jax 2019), and where it is meaningful to measure this flow to provide information to the society (Czúcz et al. 2020). This flow starts from the ecosystems, which display a number of different characteristics (e.g. biophysical structures or processes, level 1 of the cascade). These characteristics can then determine the capacity of the studied ecosystem to deliver a specific service (level 2). Depending on human inputs (e.g. harvest efforts), this capacity is then realized as an ‘actual use’, i.e. the amount of the ES that humans actually consume or use (level 3). Finally, this ES flow will result in an increase in human well-being (level 4) (Fig. 1).

‘Flood regulation’ is a term that incorporates different biophysical processes (ecosystem functions) (Crossman et al. 2019). The terminology is mainly the result of the dominant anthropocentric viewpoint of ES which reflects a human-benefit perspective of flood damage or flood protection. It does not distinguish between the various processes and factors that lead to inundations, nor the ways in which ecosystems can interact with these processes thus reducing the severity of floods (the ecosystem functions, Haines-Young and Potschin 2010). If the contribution of natural systems is to be assessed or mapped, these underlying processes will become very relevant: it is impossible to consistently measure and map nature’s contributions to flood protection without distinguishing the underlying main mechanisms.

Basically, there are two different ways ecosystems can provide flood regulation. One takes place in the whole catchment and can be called water retention, runoff regulation, incipient flood regulation or flood prevention (e.g. Nedkov and Burkhard 2012; Czúcz et al. 2018a, b; Crossman et al. 2019), which we call ‘flood prevention ES’ hereafter. Vegetation can retain a high amount of water through interception by leaves (canopy and litter), thus reducing the amount of precipitation reaching the soil surface to a considerable degree (Clark 1987; Breuer et al. 2003; European Environment Agency 2015; Zagyvai-Kiss et al. 2019), while roots increase infiltration into the soil and thereby reduce surface runoff in quantity and speed, prior to the accumulation of a flood wave (Lange et al. 2013; Zhang et al. 2014).

Once the water has accumulated into streams and rivers (or wetlands and lakes) it is mainly the ecosystems adjacent to these downstream water bodies that can interact with the flowing water, and only in a much more limited way, after relevant processes in the catchment have taken place. We will call this process ‘flood mitigation ES’ hereafter, while others also use terms like: posterior flood regulation, peak flow reduction, flood mitigation (e.g. Czúcz et al. 2018a, b; Crossman et al. 2019). In this case the adjacent ecosystems, the floodplains, only mitigate the effects of floods by offering “space” (storage capacity) for floodwater, thus reducing peak height and severity of the flood wave, but also by influencing—usually reducing—the conveying capacity of both the main channel and the floodplain. This way riparian ecosystems affect the spatial–temporal evolution of the flood waves (and thus the flood risk) in a complex way (Leyer et al. 2012).

**Fig. 2:**
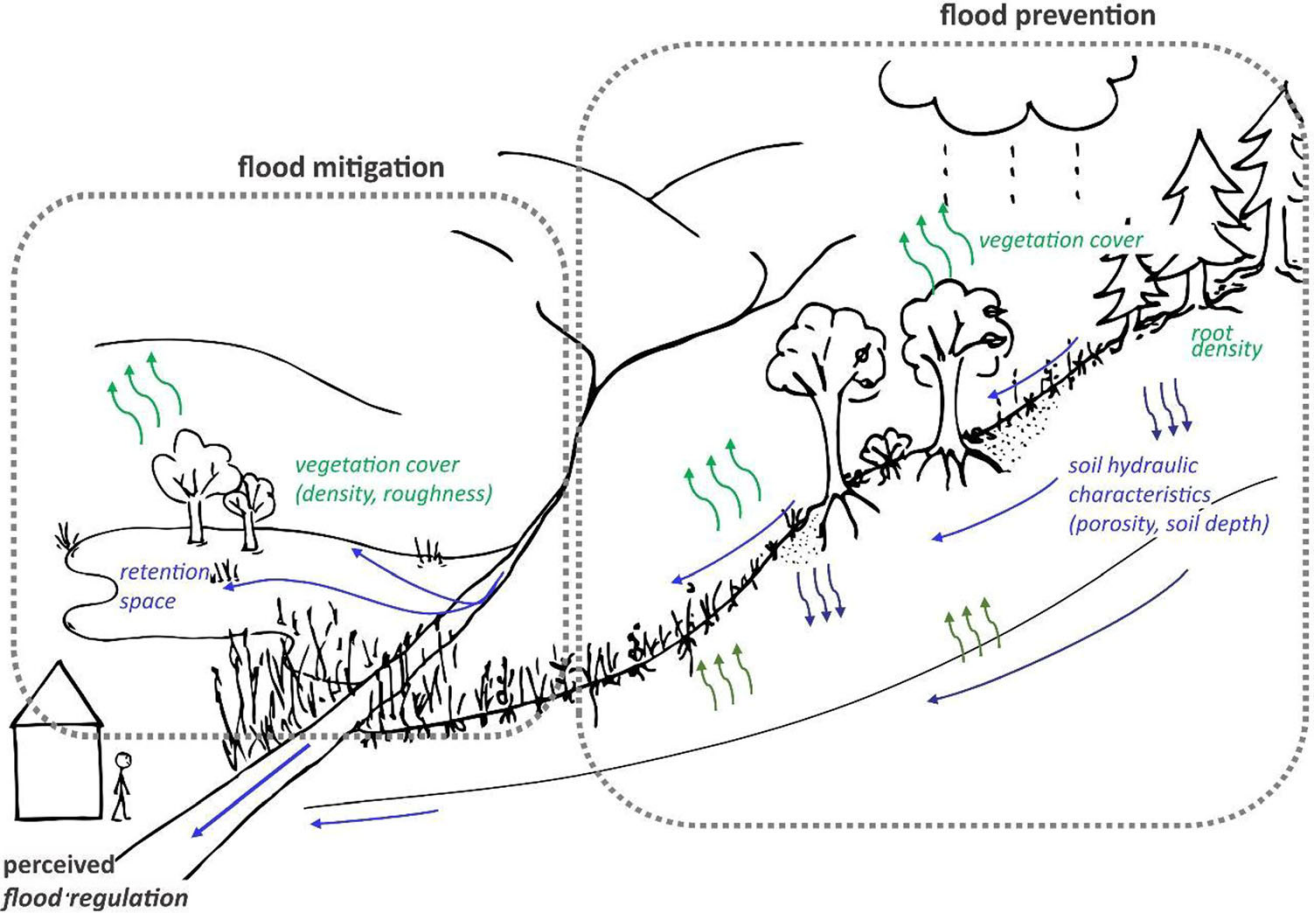
Two different mechanisms of flood regulation: ‘prevention’ in the whole catchment, and ‘flood mitigation’ along the streams in the floodable areas with relevant ecosystem characteristics (*green italics* for vegetation related, *blue italics* for abiotic), ecosystem functions (mechanisms) (bold), and human perception of ecosystem services (*bold italics*)

The underlying mechanisms are very different in the two cases, and consequently the characteristics (= condition aspects; see Box 1) of the ecosystems (as service provisioning areas) that determine the ‘efficiency’ of flood regulation will also be very different (Fig. 2).

These two ‘flood regulation’ mechanisms take place in a terrestrial context, and refer to freshwater ecosystems (rivers mainly, but lakes and wetlands basically work the same way). In a coastal context, the mechanisms that can lead to floods are highly different as they are more related to weather events other than precipitation (e.g. wind and storms), or even, non-meteorological processes (tidal waves, tsunamis). Furthermore, they are not interpreted at the catchment scale; so the contributions of coastal ecosystems to the mitigation of coastal floods can also be considered a different service (see Flood regulation in the different ES classifications section).

For ensuring a balanced functioning of the landscapes, reconciling multiple aspects and specifically so for maintaining the basis for their flood regulation capacity, we need to review which characteristics of the ecosystems are relevant for this specific ES.

Ecosystem condition is the overall quality of an ecosystem measured in terms of its abiotic and biotic characteristics which underpin the ecological integrity of the ecosystem (based on Keith et al. 2020 and UN 2021; see Box 1). It aims to measure the biophysical properties that underpin services (Schröter et al. 2016; Czúcz and Condé 2017). In contrast to the conservationist approach of evaluating and monitoring a general state of nature which is to be maintained in good condition for the sake of itself (inherent value), this definition relies on an instrumental perspective, where only those characteristics that influence the delivery of ecosystem services are considered.

A multitude of different EC aspects can be listed, which are notoriously difficult to put into a common frame as documented in several conceptual papers (e.g. Müller 2005; Kandziora et al. 2013; Roche and Campagne, 2017; Haase et al. 2018); systematic reviews (e.g. Smith et al. 2017; Rendon et al. 2019); or policy reports (e.g. Maes et al. 2018). Noticing this, the upcoming Ecosystem Accounting standards published under the UN System of Economic Environmental Accounts (SEEA EA, United Nations 2021) include a flexible Ecosystem Condition Typology (Czúcz et al. 2021), which has also been recently endorsed as a statistical standard as part of the System of Environmental-Economic Accounting—Ecosystem Accounting (SEEA EA).

There were several reviews in recent years discussing aquatic ES, including flood regulation. Nonetheless, most of these include neither the EC aspects, nor the hydrologic processes within the catchment contributing to the ES of flood regulation (Hanna et al. 2018; Kaval 2019). Some works did explore the relevant condition of rivers and lakes (e.g. Water Framework Directive related EC indices, (Vidal-Abarca et al. 2016), specifically for large river floodplains (Erős et al. 2019), or even focusing on flood regulation in floodplains (Grizzetti et al. 2019), but did not take into account the whole catchment (but see Crossman et al. 2019). The reviews focusing on the hydrological aspects were mostly on modelling (hydrological models, their suitability, parameterization, etc.) and did not involve EC aspects (apart from the spatial representation of different ecosystems and their extent). They rather tested the suitability of the models, involving validation with gauged data (Stürck et al. 2014; Redhead et al. 2016). Work on linkages between ES and EC is generally very much focused on the relationship between ES and specifically biodiversity, as one component of EC (Teixeira et al. 2019 for aquatic; van der Plas 2019) and are often not validated with biophysical data, but rely on expert assessments only (Teixeira et al. 2019).

In this paper we will:

- critically review the main ES classification systems used in ES assessments and accounting if and how they distinguish the two main types of riverine flood regulation;
- give a structured overview of which ecosystem (condition) aspects are needed for securing the delivery of the two types of the ES ‘flood regulation’
- discuss some aspects of operationalizing the assessment of flood regulation as an ES.

Box 1 Key concepts of ecosystem service assessments, as used in the paper **Ecosystem condition (EC):** the overall quality of an ecosystem measured in terms of its abiotic and biotic characteristics which underpin the ecological integrity of the ecosystem (based on Keith et al. 2020 and UN 2021)

**Ecosystem characteristic:** attributes, or ‘condition aspects’ of an ecosystem describing its components, structure, processes, and functionality. The term characteristics is intended to be able to encompass all of the various perspectives taken to describe an ecosystem (based on UN 2014 and Czúcz and Condé 2017)

**Ecosystem condition characteristic:** those ecosystem characteristics that fit the concept of ecosystem condition (based on Keith et al. 2020 and UN 2021). Relevant characteristics that do not fit the selection criteria are called ancillary data (e.g. stable environmental characteristics, see Czúcz et al. 2021). Ecosystem condition characteristics are also often called ‘condition aspects’ (Maes et al. 2018)

**Ecosystem service (ES):** the contributions of ecosystems to benefits obtained in economic, social, cultural and other human activity (Czúcz and Condé 2017)

**Indicator** (of an ecosystem service or a condition characteristic): a concrete quantitative metric which reflects a condition characteristic or an ecosystem service (or any other phenomenon of interest, based on Heink and Kowarik 2010 and Potschin-Young et al. 2018)

## Methods

In order to overcome the limitations of previous reviews and to synthesize knowledge documented in primary research studies in a systematic way, we overviewed the factsheets which were produced as part of the European Mapping and Assessment of Ecosystem Services (MAES) process supporting background work (Czúcz and Condé 2017; Czúcz et al. 2018a, b) and complemented this with experience from the work of a national expert group of an EU member state (Hungary) implementing the mapping (Kovács-Hostyánszki et al. 2019). While the MAES process provides mainly theoretical information relying on literature reviews (compiled with a strong ES view), the national expert working groups’ expertise is in hydrology. Evaluating literature and synthesizing from both perspectives is bound to result in a much more consistent ordering of the different aspects with a deeper understanding of the underlying processes. While the present work relies on a number of strictly and systematically reviewed literature items, its value is greatly enhanced by discussions with experts in the related fields validating and helping to interpret the findings. On the other hand, analysing a set of literature and providing it as a baseline for the evaluations given by experts helps streamlining valuations. This paper amalgamates the results of both approaches to develop the best possible framework for the application in integrated water management and ES based management.

### ES classification systems

In order to put the ecosystem function ‘flood regulation’ into relation with different ES classification systems, we analysed the relevant items found in the major classification systems. The following were analysed for all possible formulations of ‘flood regulation’:

- CICES 5.1 (Haines-Young and Potschin 2018)
- Nature’s Contributions to People (NCP) from IPBES (Pascual et al. 2017)
- TEEB (The Economics of Ecosystems and Biodiversity—TEEB 2010)
- MA (Millennium Ecosystem Assessment 2003).

### Reviewing ecosystem characteristics

To identify the ecosystem characteristics that can influence the capacity of the ecosystems, to supply flood regulation we conducted a focused systematic review. We modified a previously applied search strategy (Smith et al. 2017) somewhat, so that only primary research results targeting at least one of the two specified flood regulating ES were included.

- We added to the originally used search terms “flood*”, “water flow regulation”, “peak flow”, “attenuation”, “storage”, “protection”, “defence”, “prevention”, “runoff”, “evapotranspiration”, and infiltration” the terms “mitigation”, “floodplain” and “regulation” identified as relevant by the MAES-HU expert group in order to cover a broad spectrum of aspects and service specific literature.
- We only kept studies that test and document at least one relationship among an EC indicator and an ES indicator.
- Papers exclusively focusing on coastal flood protection were excluded, as well as papers that focused solely on total water yield (ES of water provision) rather than time-dependent changes in water yield.
- We only kept studies that were performed in Europe.

We continued the search until we had 10 papers for each of the two ES sub-types to have a balanced picture of both relationships. Due to the very strict rules set up, this could be only achieved by a very intense search especially for the mitigating type of flood regulation, where papers including any kind of EC indicators were rather scarce. Therefore, we regard this review, although small in the number of included items, as sufficiently thorough. The preliminary results of this work were presented in Czúcz et al. (2017, 2018a, b).

We derived from the reviewed studies the (positive, negative or mixed) relationship between ecosystem characteristics and one of the two examined ecosystem functions. For structuring the results of the systematic review we applied the SEEA ECT typology (Czúcz et al. 2021) to classify the ecosystem characteristics and their indicators (Table 1). We also grouped the relationships found according to the direction of the relationship and assigned the ecosystem characteristics to the ecosystem types that they characterized in the original studies using the MAES ecosystem typology (Maes et al. 2013) adding one additional type, ‘floodplains’ in order to reflect all ecosystem types within reach of regular flooding. For the interpretation of the relationships between the ECs and the flood regulation functions as ‘positive’ or ‘negative’, we interpreted each EC as changing into a positive direction, e.g. the attribute getting higher, bigger, larger coverage, more frequent.

**Table 1:** Typology used for classifying ecosystem characteristics (based on the SEEA EA Ecosystem Condition Typology, Czúcz et al. 2021)

*Abiotic ecosystem characteristics*		
A1	Physical state characteristics	Physical descriptors of the abiotic components of the ecosystem(e.g. soil structure, water availability)
A2	Chemical state characteristics	Chemical composition of abiotic ecosystem compartments (e.g. soil nutrient levels, water quality, air pollutant concentrations)
*Biotic ecosystem characteristics*		
B1	Compositional state characteristics	Composition / diversity of ecological communities at a given location and time (e.g. presence / abundance of key species, diversity of relevant species groups)
B2	Structural state characteristics	Aggregate properties (e.g. mass, density) of the whole ecosystem or its main biotic components (e.g. total biomass, canopy coverage, chlorophyll content, annual maximum NDVI)
B3	Functional state characteristics	Summary statistics (e.g. frequency, intensity) of the biological, chemical, and physical interactions between the main ecosystem compartments (e.g. primary productivity, community age, disturbance frequency)
*Landscape-level characteristics*		
C1	Landscape characteristics	Metrics describing mosaics of ecosystem types at coarse (landscape, seascape) spatial scales (e.g. landscape diversity, connectivity, fragmentation)
*Ancillary data*		
EE	Ecosystem extent	The area/cover/share of the main ecosystem types (in the landscape). In most cases EE has a trivial influence on ES, so generally we do not record EE (but it can be, e.g. if explicitly emphasized by the paper)
MA	Natural resource management	Ecosystem management (grazing, felling, fishing, agriculture…) characterized with its intensity

According to Czúcz et al. (2021), we do not regard the extent of ecosystem type or the management type as EC indicators, but as ‘ancillary’ data*.* However, as much of the literature reviewed focuses on these features, and, at the same time, they might be very well of relevance for management decisions, we do note them in the review tables (Tables 3 and 4).

### Expert group

During the implementation of the national MAES from 2016 to 2020 (Kovács-Hostyánszky et al. 2019), there were regular consultations with an expert group on hydrology in order to elaborate a method of how to assess the flood regulation ES at the national scale, including constraints on availability of data, time and funding. While a literature review was also part of the national assessment approach, the ES assessment itself was elaborated by a group of experts and revised by an external panel. The experts added relevant literature to the ‘factsheet’ review and assisted in the delineation of the different mechanisms behind the ES. The results from the discussions within the expert group complement the review results and provide also some special aspects for the discussion.

## Results

### “Flood regulation” in the different ES classifications

The comparative analysis of ES classifications (CICES 5.1, IPBES, TEEB and MA) showed that the two different flood regulating mechanisms of natural ecosystems, preventing and mitigating floods, were not distinguished consistently (Table 2). Actually, in most classifications even coastal floods are included under the same category. Neither TEEB nor MA make any distinction, they only relate in general terms to ‘extreme events’ or ‘hazards’ which are to be regulated by ecosystems. CICES introduces a slight distinction only towards coastal functions (by naming ‘flood control’ and ‘coastal protection’ as two ‘sub-types’ of this service). In IPBES taxonomy, flood regulation is split across two NCPs (Nature’s Contributions to People): NCP 6 clearly focuses on riverine flood regulation by water retention, while NCP 9 seems to merge the riverine flood mitigation function with coastal flood regulation. In addition to its peak flow regulation aspects, NCP 6 (as well as the old CICES v4.3 class 2.2.2.1) relates to the maintenance of baseline flows in dry periods (‘drought mitigation’). Even though this can be conceptualized as a different benefit or ES, the underlying mechanism (water retention) is the same.

**Table 2:** Position of ‘flood regulation’ in CICES (v5.1, Haines-Young and Potschin 2018) and other major ES classification systems. (IPBES: Pascual et al. 2017; TEEB 2010; MA, 2005)

Classification	Relevant categories	Definition	Functional ES type	Other ES mentioned
CICES v5.1	Section: 2 Regulation and Maintenance (Biotic) Division: 2.2 Regulation of physical, chemical, biological conditions Group: 2.2.1 Regulation of baseline flows and extreme events Class: 2.2.1.3 Hydrological cycle and water flow regulation (including flood control, and coastal protection)	Scientific: The regulation of water flows by virtue of the chemical and physical properties or characteristics of ecosystems that assists people in managing and using hydrological systems, and mitigates or prevents potential damage to human use, health or safety Simple: Regulating the flows of water in our environment	Prevention and mitigation	Coastal flood regulation
IPBES	NCP 6: Regulation of freshwater quantity, location and timing	Regulation, by ecosystems, of the quantity, location and timing of the flow of surface and groundwater used for drinking, irrigation, transport, hydropower, and as the *support of non-material contributions* (NCP 15, 16, 17). Regulation of flow to water-dependent natural habitats that in turn positively or negatively affect people downstream, including via flooding (wetlands including ponds, rivers, lakes, swamps). Modification of groundwater levels, which can ameliorate dryland salinization in unirrigated landscapes	Prevention	Base flow maintenance (droughts), water provision
	NCP 9: Regulation of hazards and extreme events	Amelioration, by ecosystems, of the impacts on humans or their infrastructure caused by e.g. floods, wind, storms, hurricanes, seawater intrusion, tidal waves, heat waves, tsunamis, high noise levels. Reduction, by ecosystems, of hazards like landslides, avalanches	Mitigation	Coastal flood regulation; microclimate regulation, noise regulation
TEEB	Moderation of extreme events	Extreme weather events or natural hazards include floods, storms, tsunamis, avalanches and landslides. Ecosystems and living organisms create buffers against natural disasters, thereby preventing possible damage. For example, wetlands can soak up flood water while trees can stabilize slopes. Coral reefs and mangroves help protect coastlines from storm damage	Mitigation	Coastal flood regulation; mass flow control (avalanches, landslides)
MA	Water regulation	The timing and magnitude of runoff, flooding, and aquifer recharge can be strongly influenced by changes in land cover, including, in particular, alterations that change the water storage potential of the system, such as the conversion of wetlands or the replacement of forests with croplands or croplands with urban areas	Prevention	Base flow maintenance, water provision

### Condition indicators ensuring flood regulating ES

Tables 3 and 4 provide an overview on the characteristics of the main ecosystem types that were found to influence the two sub-types of flood regulation respectively. According to the scientific literature sampled in the underlying systematic review, the most relevant characteristic influencing the flood prevention ES is vegetation cover (or biomass; structural ecosystem characteristics), whereas flood mitigation ES is most influenced by the opportunities for stream water to spread out (availability of ‘retention space’, water storage capacity).

**Table 3:** Ecosystem characteristics documented to influence the ‘preventing’ type of flood regulation

ECT class (and subclass)	Ecosystem characteristic	No. of papers documenting influence		Ecosystem types	References
		Positive	Negative		
A1 Physical state characteristics	Soil porosity	3		Forest, grassland, heathland, sparsely vegetated area	Cosandey et al. (2005), Hümann et al. (2011), Lana-Renault et al. (2014)
	Soil thickness	1		Forest, grassland, heathland, sparsely vegetated area	Lana-Renault et al. (2014)
A2 Chemical state characteristics					
B1 Compositional state characteristics					
B2 Structural state characteristics	Belowground (root density)	1		Forest	Lange et al. (2013)
	Litter	1		Heathland	Schmittner and Giresse (1996)
	Aboveground cover	4		Forest, grassland, heathland, sparsely vegetated area	Robinson et al. (1991), Schmittner and Giresse (1996), Cosandey et al. (2005), Lana-Renault et al. (2014)
	Aboveground height	2		Forest, grassland, heathland, Wetland	Robinson et al. (1991), Schmittner and Giresse (1996)
B3 Functional state characteristics	Fire frequency		1	Cropland, forest, heathland, sparsely vegetated area	Aronica et al. (2002)
	Successional age	3		Forest	Robinson et al. (1991), Cosandey et al. (2005), Hümann et al. (2011)
C1 Landscape characteristics	Impervious surfaces		1	Urban	Stovin et al. (2012)
Ancillary data types	Ecosystem extent	3		Forest	Clark (1987), Cosandey et al. (2005), EEA (2015)
	Natural resource management		2	Forest, grassland, wetland	Robinson et al. (1991), Cosandey et al. (2005)

**Table 4:** Ecosystem characteristics documented to influence the ‘mitigating’ type of flood regulation

ECT class (and subclass)	Ecosystem characteristic	No. of papers documenting influence		Ecosystem types	References
		Positive	Negative		
A1 Physical state characteristics	Soil porosity	1		Floodplain, forest	Barth and Döll (2016)
A2 Chemical state characteristics					
B1 Compositional state characteristics					
B2 Structural state characteristics	Aquatic vegetation		2	Floodplain, water, wetland	Trepel et al. (2003), Bal and Meire (2009)
	Aboveground cover	1	3	Floodplain	Darby (1999), Thomas and Nisbet 2006), Habersack et al. (2009), Nagy et al. (2018)
B3 Functional state characteristics					
C1 Landscape characteristics					
Ancillary data types	Ecosystem extent	4		Floodplain, forest	Acreman et al. (2003), Castellarin et al. (2011), Barth and Döll (2016), Grizzetti et al. (2019)
	Pre-aggregated indicators	1		Water	Grizzetti et al. (2019)

These tables also specify characteristics of relevance for each ecosystem type. Flood prevention ES take place in all terrestrial ecosystem types (highly depending on their characteristics), whereas flood mitigation only involves rivers, lakes, wetlands and all floodable terrestrial ecosystems (floodplains) adjacent to them. The latter might include numerous different ecosystem types, nevertheless we introduced a special ecosystem type in our tables for representing these flood-prone (mostly natural), non-built up areas.

### Expert group additions

The expert group on hydrology emphasized the importance of some factors that are not EC aspects according to SEEA (Czúcz et al. 2021), but nevertheless of great value as a rough guide for assessments.

For the flood preventing function, a feature that is not already reflected in Tables 3 and 4 is topography, or rather its influence on flow accumulation. The expert group agreed on using the topographic wetness index as a proxy in order to represent local runoff processes.

Soil hydrologic attributes were also chosen by the experts to characterize ecosystem characteristics relevant for the preventing flood regulation. If those properties are regarded, which can be modified, e.g. as an effect of good/bad (agricultural) management, it qualifies as an EC indicator.

For mitigating floods in floodplains, experts agreed on the outstanding importance of available space in the floodable area (retention space). For operationalizing this characteristic, calculating the ratio between the water volume exceeding the flood protection capacity and the available storage volume was suggested.

## Discussion

In order to be able to respond to increasing flood risks, an integrated approach is needed, which covers the relevant aspects of human benefits while also taking into account the different processes within the whole catchment. In the following sections, we discuss how the ecosystem characteristics presented—based on both literature and expert opinion—can be interpreted in terms of usefulness for modelling and for implementing flood protection, as well as conservation targets.

### Different mechanisms but the same ES class

We differentiated two ecosystem functions, two mechanisms how riverine ‘flood regulation’ occurs: an incipient flood regulating function preventing floods, and a posterior flood regulation mitigating already accumulated floods in the floodplain.

For the prevention function the structural characteristics of the source ecosystems (e.g. vegetation and surface texture) are of key importance, determining the capacity of the local ecosystem to retain some water and slow down runoff. The mitigation function is primarily governed by the geometrical / morphological characteristics of the broader riverbed, or the whole floodplain, thus it is much less affected by the ecological or biotic characteristics of the ecosystems themselves. Based on this fundamental difference, we distinguish these two functions in the following discussion as two ‘sub-types’ of the studied ES.

Despite the differences of the underlying mechanisms and contributing ecosystems, most of the widespread ES classifications do not reflect these differences. The fact that the two most used ES classifications TEEB and partly also CICES seem to be insensitive to these differences might be due to categorizing the ES from the beneficiaries’ perspective. From this perspective all these cases appear as the same type of ‘water damage’ (avoided or not). Put within the ecosystem services framework, the two different functions can be linked to the ES cascade level 2, while the ES classifications grasp only cascade level 3–4 reflecting on the benefits and well-being humans receive.

### Ecosystem characteristics for the two sub-types

We found several characteristics named in the literature as enabling the two different ecosystem functions resulting in the attenuation of floods. Not all of them were consistent with the selection criteria for EC according to the SEEA. In the following we reflect on the major characteristics and their usefulness as EC indicators, adding insights resulting from discussions within the hydrology expert group.

#### Characteristics influencing the preventing type of flood regulation

Structural characteristics, often as ‘vegetation cover’, but sometimes simply as ‘biomass’ were one of the most frequently mentioned type of characteristics. It represents the structural elements of ecosystems that intercept precipitation and unite all other (biotic) functions important for water retention within a catchment (infiltration, transpiration, increased surface retention) (Robinson et al. 1991; Schmittner et al. 1996; Cosandey et al. 2005; Stovin et al. 2012; Lana-Renault et al. 2014). For one, the amount of biomass or vegetation cover, can be used to circumscribe an ecosystem type (in terms of delineating one ecosystem type from the other based on its biomass, e.g. grasslands vs. forests), and re-occurs in every study, emphasizing the share of vegetated areas, specifically forests within a catchment. But as an EC indicator, it can be also interpreted within each ecosystem type as one feature of that type reflecting an ecosystem quality that enhances the ES flood regulation. Several characteristics we found also alter the capacity to retain water by changing the ecosystem type’s biomass—like human management activities and natural disturbances, such as clearcutting or fire events (Robinson et al. 1991).

A closely related aspect is the share of (im)permeable or (im)pervious surfaces, which is often expressed as vegetated vs. non-vegetated areas (Bagstad et al. 2013; Farrugia et al. 2013; Boyanova et al. 2016), and can thus be interpreted in terms of ecosystem structure (biomass, cover, vegetation height) too.

Infiltration and rainfall induced runoff are also closely linked to attributes related to soil quality (Nepstad et al. 1994; Egoh et al. 2012; Nedkov and Burkhard 2012). They present a completely different category of EC as they are not directly related to present land cover. Nevertheless, soil attributes determine the process of runoff generation to a large extent (Schüler 2006; Hümann et al. 2011), thus making the services provided by the actual land cover the more valuable the ‘poorer’ the soil is. Governing soil hydrologic attributes mainly depend on genetic soil type, but soil organic content is modifiable and generally held as a good indicator for soil health as it can influence both water retention capacity and hydraulic conductivity (e.g. Stürck et al. 2014; Parker et al. 2016; Maes et al. 2017). Even though there is a lot of research showing that management practice influences soil organic content, latter has only a limited—and often overestimated—effect on soil hydraulic properties (Minasny and McBratney 2018). At the same time, one must note that management practice can improve the soil water regime through other related processes (e.g. helping infiltration with increased surface roughness and improved soil structure or limitation of evaporative losses by shading and coverage).

#### Characteristics influencing the mitigating type of flood regulation

In contrast to the prevention function, the presence of biomass or vegetation cover (structural characteristics) seems to be generally disadvantageous for the mitigation function of regulated rivers, as dense vegetation increases floodplain roughness, and slows down flood conveyance in the floodplains (Morris et al. 2005; Doherty et al. 2014; Nagy et al. 2018). However, quick runoff from one river segment can increase flood risk in another segment, therefore, slowing down a flood wave, especially upstream before settlements, can be also seen as positive (Habersack et al. 2009; Thorp et al. 2010; Barth and Döll 2016; Schober et al. 2018; Crossman et al. 2019).

Unlike for the prevention flood regulation, for (rivers and) floodplains, biomass has a controversial role: a reduced biomass or vegetation cover can expose artificial defence measures (e.g. dykes) more to the force of flood waves, wave erosion and ice drift but at the same time it also means reduced roughness and thus faster conveyance. Furthermore, dense and especially woody vegetation cover is accompanied by an extensive root system and favours wildlife and animal burrowing. These biotic effects can significantly compromise the structural integrity of the dams and can lead to hydraulic failure of protective earthworks. Thus, the presence of the vegetation on or near the flood protection levees and in the floodplain is a complex and controversial question (Corcoran et al. 2010; Bayoumi and Meguid 2011; Wenger 2015).

In floodplains, the most important characteristic regarding flood risk is the presence and the amount of floodable ecosystems or retention space (Thorp et al. 2010; Barth and Döll 2016; Grizzetti et al. 2019). Wetlands, including temporarily connected side-channels, are an ideal ecosystem type for temporary flood water storage (Acreman et al. 2003; Bullock and Acreman, 2003; Pataki et al. 2013), with their storage capacity depending on the degree to which their water levels are allowed to fluctuate, in general, or more specifically, on the space that is available at the moment (Acreman et al. 2003). This is also true for the space in the soil itself, as pore space, that can store more water after a period of drought (Palmer and Smith, 2013). The connectivity between the main channel and side-arms (i.e. the floodable space) is an important feature not just for flood regulation but also for nature conservation and is incorporated in the Water Framework Directive related monitoring too (Erős et al. 2019; Hein et al. 2019).

While retention space is the most decisive in how a flood is conveyed or regulated, it rather characterizes the actual capacity of the reach in question than its condition. But even if used as an ES capacity indicator for flood regulation, we can see that due to its very physical origin (space), which is not directly connected to (biotic) features of the ecosystem, nor the ecosystem type, it is difficult to handle it consistently within an ES framework.

A useful approach might be the use of some ratio of the indicator ‘retention space’, as suggested in literature e.g. between the ‘natural’ or historic (geomorphologic) floodplains and the present floodplain that is accessible to flood water (Podschun et al. 2018; Grizzetti et al. 2019). Further suggestions from the expert group included calculating the ratio between the water volume exceeding the flood protection capacity and the available storage volume which can give a present day based assessment of the flood retention capacity of the respective reaches.

In contrast to structural flood protection infrastructure, natural flood water retention, either in naturally large floodplains, (semi-) natural reservoirs or restored wetlands, is mostly profitable due to much lower maintenance costs and far lower chances of catastrophic failure (Acreman et al. 2003; Opperman et al. 2009; Thorp et al. 2010). Nevertheless, while many restoration initiatives have started (González et al. 2015), giving more space to floods is still often not implemented in today’s water management practice. Other ecosystem types in the former river floodplains—often converted to grasslands or arable land—can be also useful for temporary flooding (Acreman et al. 2003; Morris et al. 2005). Here, cost–benefit analyses or a risk-based evaluation of the different flood control options should be targeted in line with the EU Flood Directive (2007/60/EC), including an analysis of different scenarios with flooding adjacent land on former floodplains, calculating storable amount of water, flood peaks, frequency of flooding or flood risk, vulnerability of and values on the flooded areas (e.g. potential losses to agriculture) as well as costs of flood defence (infrastructure + operational flood management), but also including positive side-effects of prolonged water storage and nutrient enrichment of soils, enhanced groundwater recharge, maintenance of base flows (Kron 2005; Gallay and Olah, 2017; Crossman et al. 2019). Within an ES framework, it is more helpful to resort to scenarios including ‘potentially floodable areas’ or ‘if the whole floodplain were left natural’ (as in Albert et al. 2015, or Grizzetti et al. 2019), than assessing solely the potential ES of the area that is actually floodable at the present conditions, as these are strongly limited in most of Europe. By assessing different outcomes in a risk-based approach, choosing the option with the ‘minimized sum of incurring and/or mitigating the damage’ offers a solution towards a more integrated water management strategy that can be very well brought in alignment with the ES concept (Crossman et al. 2019).

## Conclusion

Reviewing the relevant ecosystem characteristics of the two ES (sub-types ‘preventing’ water accumulation to a flood and ‘mitigating’ an already accumulated flood wave) showed that both are influenced mostly by the physical properties of the ecosystem types (e.g. biomass, soil), while biodiversity is not really relevant for supporting these two services. Nevertheless, biodiversity is important from several other aspects and might be also related in more indirect ways to flood regulation that are not apparent from this review. From the two ES, it is ‘flood prevention’ that can be more naturally integrated into ES assessments, as it gives a service to all downstream beneficiaries. The ‘mitigation ES’, on the other hand, is a spatially complex, relative process, where saving one place from floods can create flooding in other locations.

Recognizing the hydrologic functions behind what we defined as the ‘preventing’ type of flood regulation ES gives the basis for valuating this ES at the appropriate scale, including the whole catchment. It also enables the delineation of potentially critical service providing areas—those areas that are especially important for maintaining the ES, or that might be of priority for restoration. The preventing ES seems to be about three times more frequently assessed as an ES (based on the sample taken by Smith et al. 2017)—which might underline the fact that it is more straightforward to conceptualize as an ES, and fit into a MAES context.

On the other hand, the ‘mitigating’ type of flood regulation, that takes place within the floodplains, challenges the interpretation and application within an ES framework, as it is spatially more complex and context dependent. In the specific hydrologic literature there is a vast amount of flood modelling studies; however, these are less commonly framed as ES studies. Integration of the flood risk concept (cf. EU Flood Directive) can forward effective decision making based on a cost–benefit principle into which assessed ES components can be included.
